# Effect of Vertically Propagating Shear Waves on Seismic Behavior of Circular Tunnels

**DOI:** 10.1155/2014/806092

**Published:** 2014-01-30

**Authors:** Tohid Akhlaghi, Ali Nikkar

**Affiliations:** Faculty of Civil Engineering, University of Tabriz, Tabriz 5166116471, Iran

## Abstract

Seismic design loads for tunnels are characterized in terms of the deformations imposed on the structure by surrounding ground. The free-field ground deformations due to a seismic event are estimated, and the tunnel is designed to accommodate these deformations. Vertically propagating shear waves are the predominant form of earthquake loading that causes the ovaling deformations of circular tunnels to develop, resulting in a distortion of the cross sectional shape of the tunnel lining. In this paper, seismic behavior of circular tunnels has been investigated due to propagation of shear waves in the vertical direction using quasi-static analytical approaches as well as numerical methods. Analytical approaches are based on the closed-form solutions which compute the forces in the lining due to equivalent static ovaling deformations, while the numerical method carries out dynamic, nonlinear soil-structure interaction analysis. Based on comparisons made, the accuracy and reliability of the analytical solutions are evaluated and discussed. The results show that the axial forces determined using the analytical approaches are in acceptable agreement with numerical analysis results, while the computed bending moments are less comparable and show significant discrepancies. The differences between the analytical approaches are also investigated and addressed.

## 1. Introduction

The response of underground structures to dynamic loading is significantly different from that of above ground structures. Underground structures do not fall in resonance with the ground, however, response in accordance with the response of the surrounding ground. Therefore, seismic design loads for underground structures are generally characterized in terms of the deformations imposed on the structure by the surrounding ground. The free-field ground deformations due to a seismic event are estimated, and the tunnel is designed to accommodate these deformations. Underground structures are assumed to experience three primary modes of deformation during seismic shaking [1]:

1. axial compression and extension;
2. longitudinal bending;
3. ovaling/racking.

**Figure 1:**
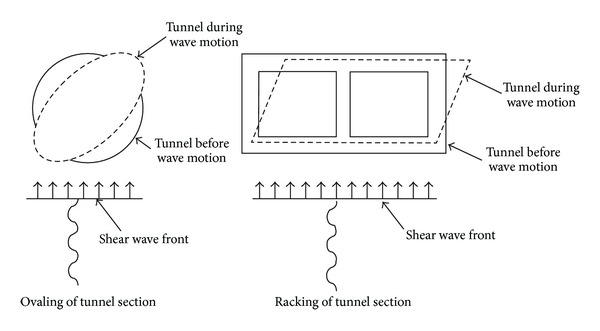
Ovaling and racking deformation modes of tunnels.

Axial deformations in tunnels are generated by the components of the seismic waves that produce motions parallel to the axis of the tunnel and cause alternating compression and tension. Bending deformations are caused by the components of seismic waves producing particle motions perpendicular to the longitudinal axis. Design considerations for axial and bending deformations are generally in the direction along the tunnel axis [2]. Ovaling or racking deformations in a tunnel structure develop when shear waves propagate normal to the tunnel axis resulting in a distortion of the cross sectional shape of the tunnel lining. Design considerations for this type of the deformation are in the transverse direction; see Figure 1. The general behavior of the lining may be simulated as a buried structure subject to ground deformations under a two-dimensional plane-strain condition.

Studies have suggested that, while ovaling may be caused by waves propagating horizontally or obliquely, vertically propagating shear waves are the predominant form of earthquake loading that causes these types of deformations [2]. There are two procedures to compute deformations and forces corresponding to the three above-mentioned deformation modes:

1. free-field deformation approach;
2. soil-structure interaction approach.

In this paper, seismic behavior of circular tunnels has been investigated due to propagation of shear waves in the vertical direction using quasi-static analytical approaches as well as numerical methods. This issue has been investigated in the past by several researchers using numerical and analytical methods, which has led to solutions and results. In particular, analytical solutions are still very popular, as confirmed by the growing body of the literature devoted to this topic: while early studies referred to simplified geometries (i.e., circular tunnel, lining schematised as a closed ring, etc.) and constitutive assumptions (i.e., single-phase linear-elastic soil), recently proposed closed-form solutions deal, as an example, with piecewise tunnel lining connected by joints [3], nonuniform lining thickness [4], and poroelastic fully saturated medium (e.g., [5–8]). Some analytical solutions are based on simplified uncoupled approaches: a free-field seismic ground response analysis is first carried out and the resulting maximum displacements are then applied to a static model of the underground structure. For a synopsis of the available uncoupled solutions, the reader can refer to St. John and Zahrah [9], Penzien and Wu [10], and Wang [2]. Many real tunnel projects are characterised by relatively complex conditions in terms of heterogeneity of the soil strata, nonregularity of the tunnel geometry, preexistence of surface and subsurface structures, and groundwater flow. In such cases, the analysis of the seismic behaviour of the underground structure can take advantage of the use of numerical methods, like the boundary element method (BEM) (e.g., [11, 12]) and the finite element method (FEM) (e.g., [13–15]).

The surrounding soil of the tunnels constructed in the urban areas, usually consisting of the alluvium and low strength soil, experiences large deformation during ground motions. In this case the soil is voided elastic state and undergoes large plastic deformation. One of the best methods for modeling large deformations is finite difference method. The use of structural elements available in FLAC allows the dynamic soil-structure interaction analysis to be performed. In this research the nonlinear finite difference method has been used, which allows the dependence of damping and stiffness of the material to the strain level to be considered and taken into account. Also, the shallow circular tunnels under the propagation of shear waves have been analyzed to compare the analytical solutions with the numerical finite difference method. The geotechnical properties of the materials used in the analyses are obtained from the geotechnical investigations performed for the second line of Tabriz urban subway project (Iran). Also the input variables such as the maximum shear strain at tunnel's level, the shear modulus proportional to the level of the strain, and the maximum particle velocity of the mass required in the analyses with the analytical methods are determined using two-dimensional free-field numerical analysis.

## 2. Cross Section's Properties

In order to make use of actual data, the cross sections and their material properties chosen for the analyses are obtained from the geotechnical reports prepared for Tabriz subway project. The properties have been used from the geotechnical reports including the reports of geophysics vibration tests to determine the elastic properties for dynamic analysis and the goal of these tests is measuring the velocity of volume and shear waves in cross sections to determine the dynamic properties of the soil. The data and specifications of Sections 1 and 2 used in the analyses are shown in Table 1.  Table 2 also shows the layers properties of those sections obtained from project geotechnical reports.

**Figure 2:**
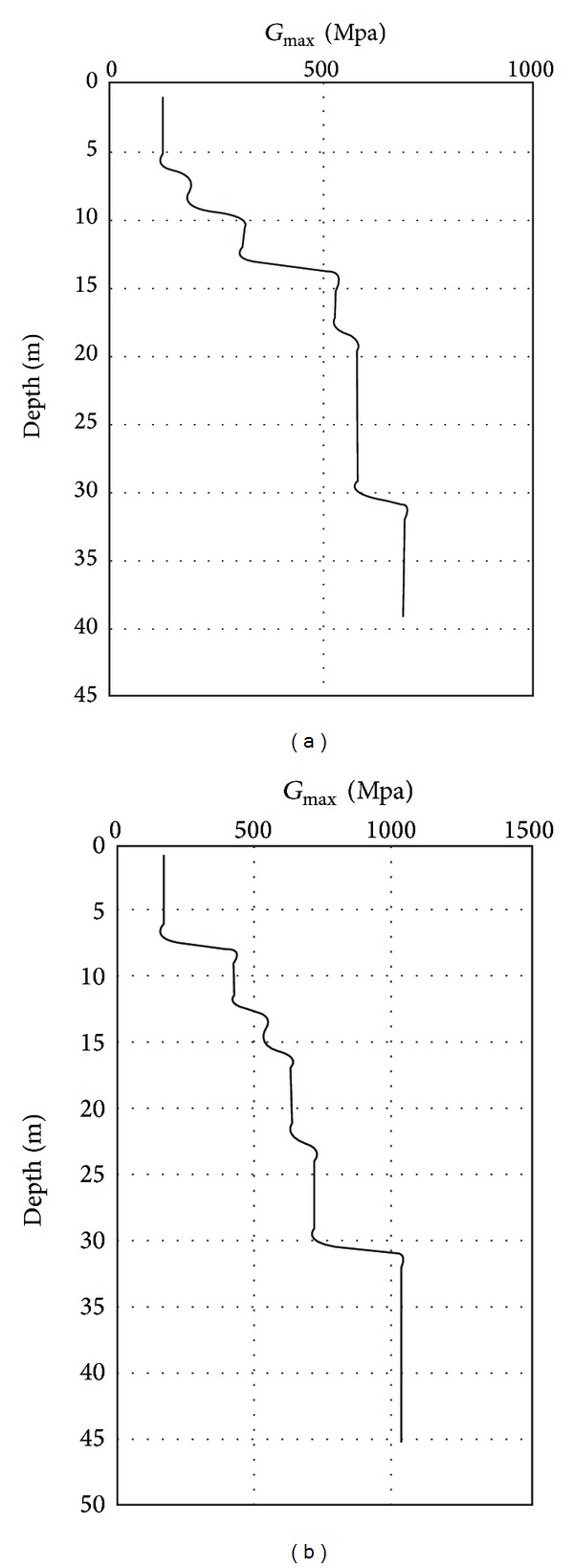
Maximum shear modulus variation with depth: (a) Section 1 and (b) Section 2.

**Figure 3:**
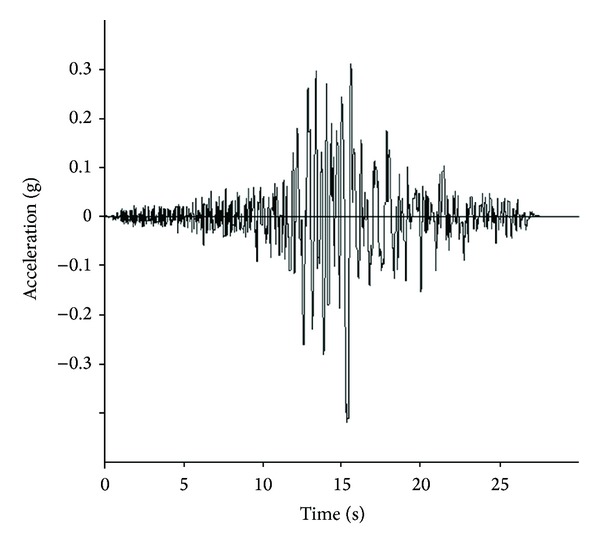
Acceleration record of the Landers earthquake.

Figure 2 shows the variation of the maximum shear modulus with depth at the site obtained from the geophysical vibration methods carried out in the project area. Also in this research, the ground motion data have been chosen according to the seismic properties of the site. The Landers earthquake (1992/6/8) ground motions data is selected to provide the expected seismic information required for dynamic analyses, as there are appropriate similarities between the two region seismotectonic characteristics; see Figure 3.

Also shown in Table 3 are the tunnel lining properties used in the analyses.

## 3. Numerical Analysis

The numerical analyses were performed with the code FLAC [16] which allows for the full nonlinear dynamic analysis. The dynamic analysis is based on the nonlinear finite difference method. The use of structural elements available in FLAC allows a dynamic soil-structure interaction analysis to be performed. The nonlinear stress-strain behavior of the soil can be simulated with nonlinear models which follows the stress-strain path during the seismic loading. The proposed nonlinear models are defined by employing the following two steps:

1. determination of the backbone (skeleton) curve;
2. application of a set of rules relevant to behavior of loading and unloading and reduction of stiffness along with other required parameters.

By defining the hysteretic model for dynamic analysis, the modulus reduction curve of the material is determined as a skeleton curve for conducting nonlinear analysis. For dynamic analysis, the elements dimensions are limited by the criterion of wave transmission. The dimension of the largest element and the minimum velocity of the shear wave are used to determine the maximum value of the frequency as follows:

(1) $$\begin{array}{c} f=\frac{C_{s}}{10\Delta l}, \end{array}$$

where *C*_*s*_ is the minimum velocity of the shear wave and Δ*l* is the largest dimension of element. For each cross section the maximum frequency is determined and, by use of the code filtration function, the higher frequencies are deleted from the ground motion data.

The lateral boundaries of the model must take into account the free-field conditions. In order to minimize the reflection of waves from bottom boundary of the model, the boundary is considered as quite boundary, and consequently the seismic input should be defined as a stress wave. By integrating the acceleration time history, the velocity time history is determined and the input wave is subsequently changed into the stress shear wave using the following relationship:

(2) $$\begin{array}{c} \sigma_{s}=2\left(\rho {\text{C}}_{s}\right)v_{s}, \end{array}$$

where *ρ* is the density, *σ*_*s*_ is the applied shear stress, *C*_*s*_ is propagation velocity of the shear wave, and *v*_*s*_ is shear particle velocity.

**Figure 4:**
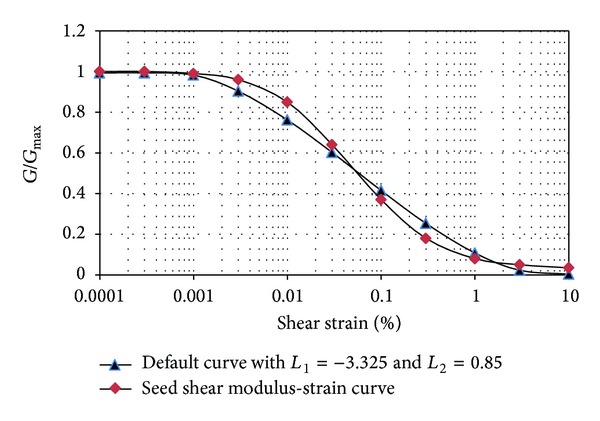
Variation of shear modulus with strain in sandy soils.

**Figure 5:**
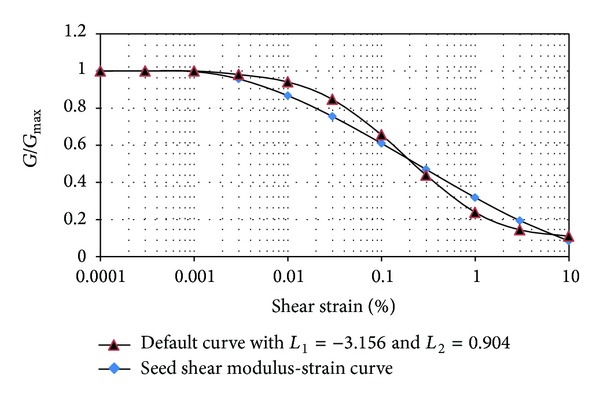
Variation of shear modulus with strain in clayey soils.

The acceleration of the top and bottom joints is also compared with the maximum input acceleration, and if needed the applied coefficient can be corrected in such a way that the modified acceleration approximates the exact value. The hysteretic damping is used to attenuate the energy of the numerical modeling. The modulus reduction curves proposed by Sun et al. [17] for fine materials and the ones proposed by Seed and Idriss [18] for coarse materials are used in this study for clays and sandy soils, respectively. As derivative of the modulus reduction curve is required in the hysteretic damping formulation, the hysteresis damping curve should be defined as a continuous curve. So the applied reduction curve should be compatible with the available functions of the used software. This curve is defined as an S shape curve in which the gradients at top and bottom strain points are zero. The following relationships for the default hysteretic model in the FLAC are used for this purpose:

(3) $$\begin{array}{c} M_{s}=s^{2}\left(3-2s\right),\\ s=\frac{L_{2}-L}{L_{2}-L_{1}},\;\;L=\text{lo}{\text{g}}_{10}\left(\gamma \right) \end{array}$$

in which *M*_*s*_ is secant modulus, *γ* is the shear strain, and L1 and L2 are bounds of the logarithmic strain (points at which gradients are zero). Figures 4 and 5 show the reduction modulus curves and the compatible function curves.

## 4. Analytical Methods

Vertically propagating shear waves are the predominant form of earthquake loading that causes the ovaling deformations of circular tunnels to develop, resulting in a distortion of the cross sectional shape of the tunnel lining. The most important and useful analytical methods for calculating these deformations are Wang and Penzien methods [2, 19] and Bobet method [20]. During 1993–2003 these methods were developed based on closed-form solutions in terms of thrust, bending moment, and displacement under external loading conditions [21–27].

### 4.1. Wang and Penzien Methods

**Figure 6:**
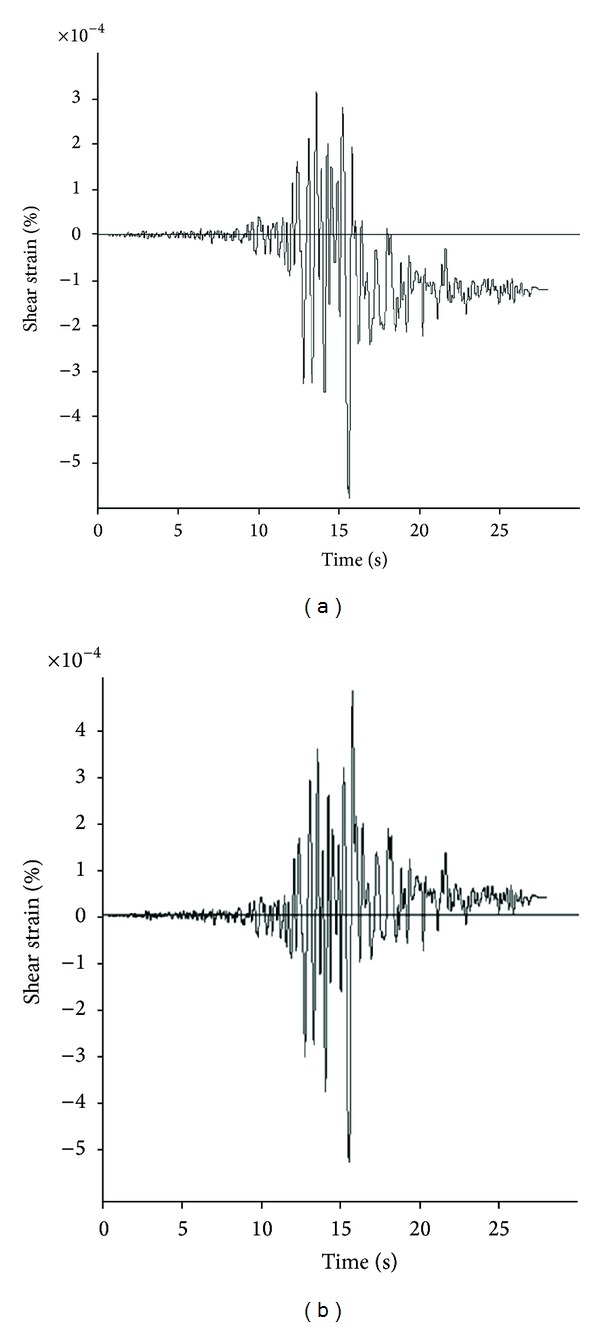
Shear strain records at tunnel axis for free-field model: (a) Section 1 and (b) Section 2.

**Figure 7:**
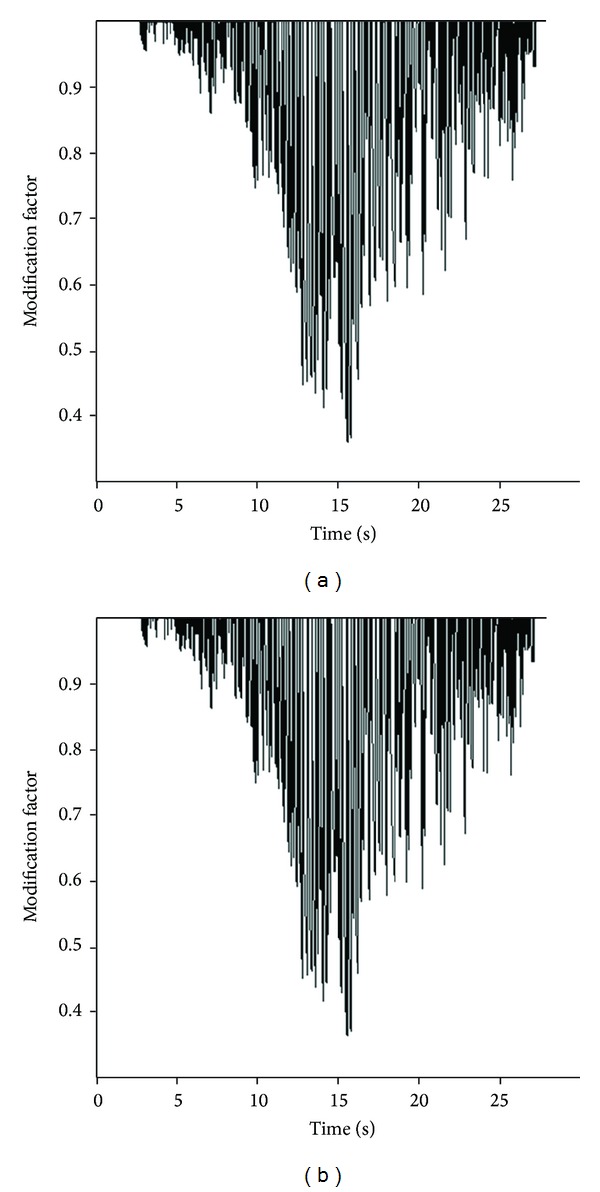
Shear modulus modification factor time histories at tunnel axis: (a) Section 1 and (b) Section 2.

Wang and Penzien solutions are developed for both full-slip and no-slip condition between the tunnel and lining. As the structural elements are directly joint to the surrounding soil, the no-slip condition is used in this paper. The input parameters in Wang and Penzien methods are cover's geometric and elastic properties, elastic properties of soil mass, and the maximum shear strain caused by the ground motions. The basic point in Wang and Penzien methods is determining the shear modulus. The shear modulus of soil materials is dependent on the level of shear strain and on higher shear strain levels; the maximum shear modulus determined by the geophysical tests will be decreased. The results obtained by use of the maximum shear modulus are therefore overestimated, and consequently the compatible shear modulus should be used. The shear modulus and shear strain parameters are determined by numerical modeling of the free-field condition. Figures 6 and 7 show the time history curves for shear strains and shear modulus reduction factors. The maximum shear strain and the reduced shear modulus are extracted from these curves.  Table 4 shows these values.

### 4.2. Bobet Method

Bobet [20] presented a new analytical method, based on the relative stiffness procedure to determine the static and dynamic loads acting on the support. The solution can account for drainage conditions at the ground-liner interface and effect of groundwater pressure on the ground and support responses. As with the relative stiffness method, it is assumed that the ground and the liner are elastic and the plane strain conditions apply at any cross section of the tunnel.

**Figure 8:**
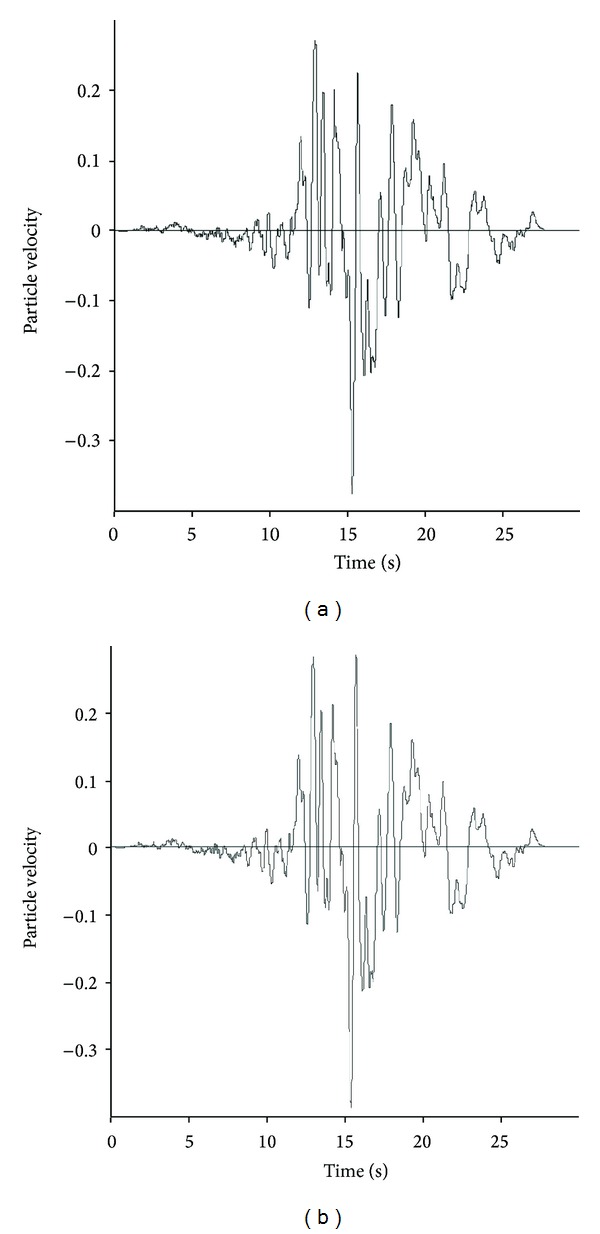
Particle velocity of joint points at tunnel axis for free-field model: (a) Section 1 and (b) Section 2.

Bobet presented the solution by assuming the no-slip condition between the tunnel and the lining at dry and saturated conditions. In this study, both conditions have been considered and investigated. The maximum particle velocity at the tunnel axis is obtained by the free-field numerical modeling.  Figure 8 shows the time history records.

## 5. Numerical Analysis

**Figure 9:**
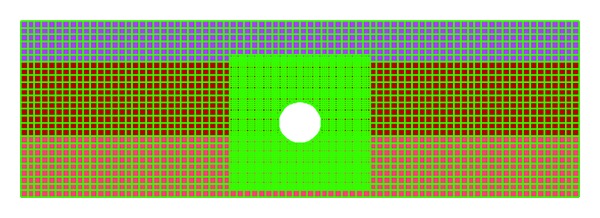
Numerical model of Section 1.

In numerical models, the tunnel cover has been shown with 72 structural elements which are rigidly connected to each other. The number of the elements is governed by the size of the surrounding zones, and therefore, in order to increase the accuracy, one structural element is created in each surrounding zone of the excavated tunnel. Dynamic loading is used in the models which are in statically stable conditions. By considering excavating conditions, the numerical models are first reached to the static equilibrium and then the dynamic analysis can be started. The numerical model of cross section 1 has been shown in Figure 9.

**Figure 10:**
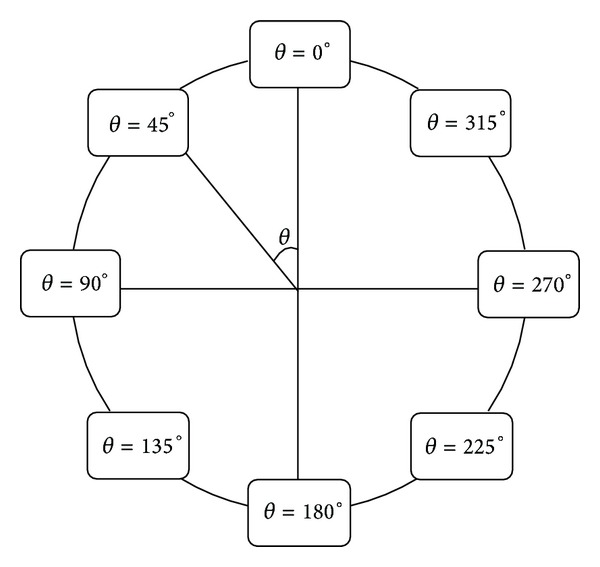
Structural elements position on the cross sections.

In this research, 8 structural elements have been chosen for each cross section in order to record the force and moment time histories during dynamic loading.  Figure 10 shows the position of these elements.

**Figure 11:**
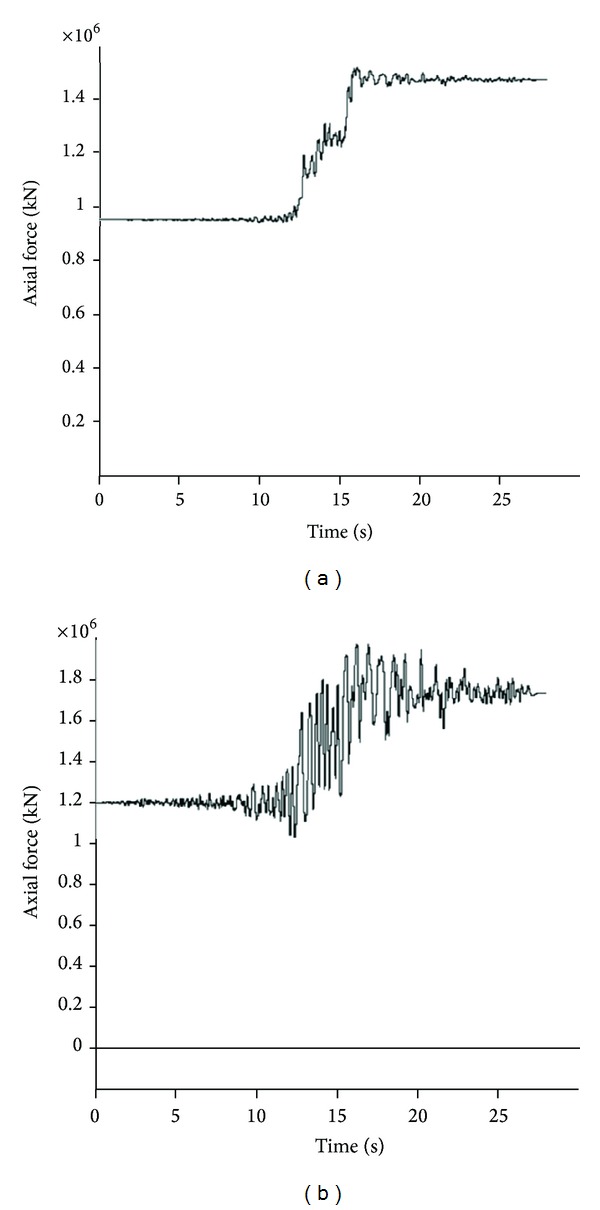
Axial force time history for Section 1: (a) *θ* = 0°; (b) *θ* = 45°.

**Figure 12:**
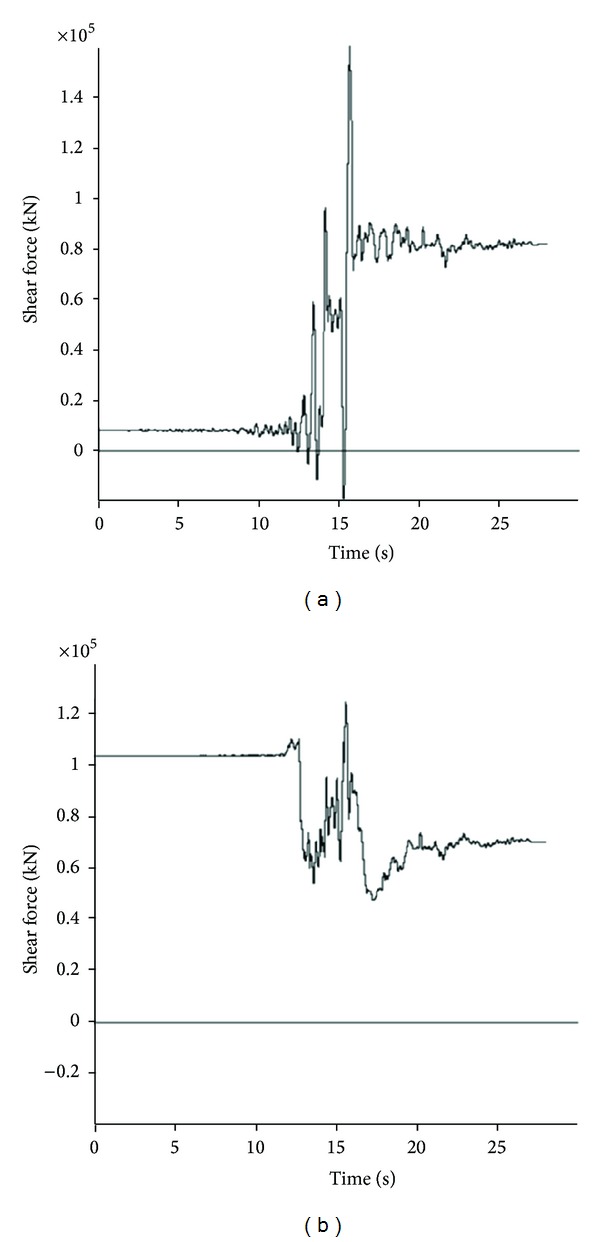
Shear force time history for Section 1: (a) *θ* = 180°; (b) *θ* = 225°.

**Figure 13:**
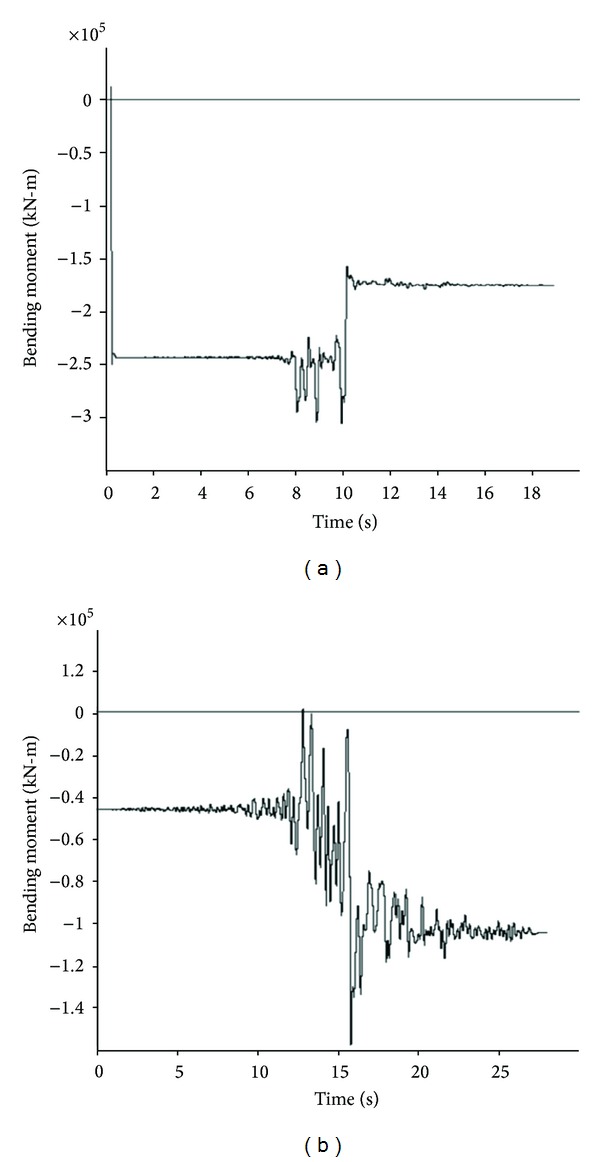
Bending moment time history for Section 1: (a) *θ* = 270°; (b) *θ* = 315°.

The results of the numerical analyses are obtained by determining the moment and force time histories during model seismic motions along with their maximum values. In the stable condition, before applying dynamic input, values of these parameters detracted from the maximum values during the dynamic wave passage, in order to determine the net maximum values. Owing to the generation of numerous time history curves and for the sake of space saving, only two history curves for each of axial and shear forces as well as bending moments related to two points of the above-mentioned eight structural element points at various positions are provided. Figures 11, 12, and 13 show these envisaged results for Section 1.

## 6. Evaluating Results

**Figure 14:**
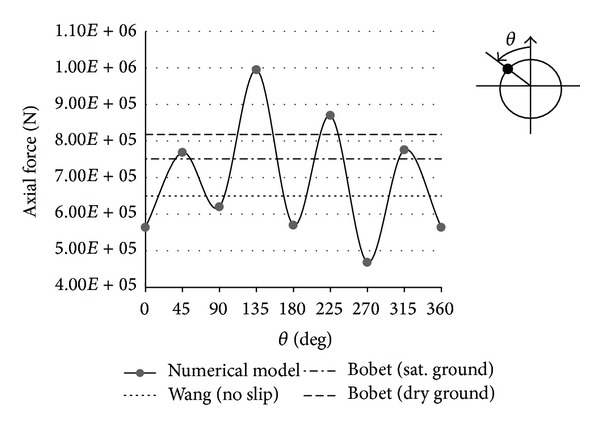
Axial forces at Section 1 due to seismic shear wave propagation.

**Figure 15:**
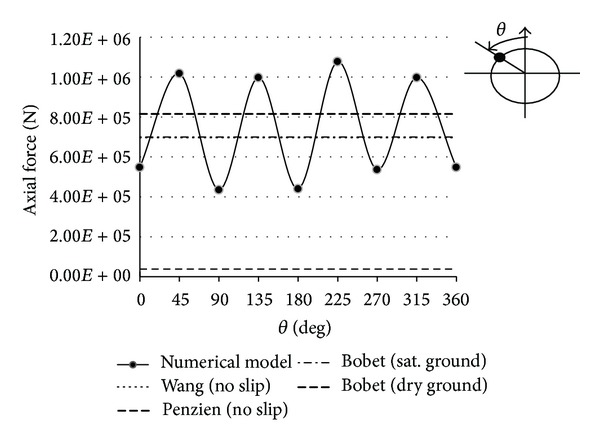
Axial forces at Section 2 due to seismic shear wave propagation.

In this section, forces imposed on tunnel lining after seismic loading are investigated. Also, results of the finite difference numerical method are compared with the results obtained by Wang-Penzien and Bobet analytical methods in the no-slip condition. One maximum value for axial force is determined by Wang and Penzien methods. Also for seismic ground motions induced by shear waves, one maximum value is obtained for axial force by Bobet method. As shown in Figures 14 and 15, results are shown by horizontal lines in the axial forces curve. Figures 14 and 15 also show the results of the analytical solutions. The maximum difference values between two results are shown in Table 5.

**Figure 16:**
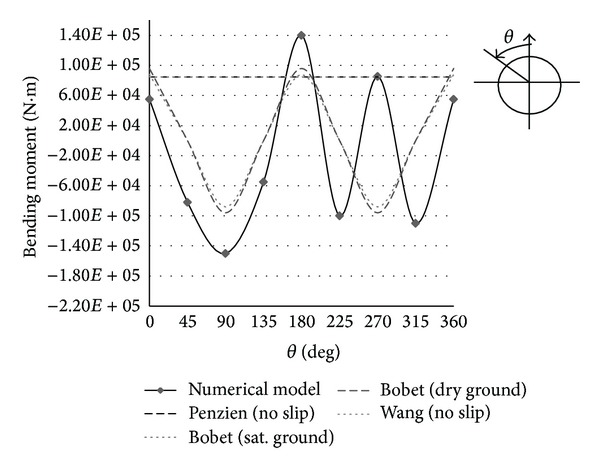
Bending moments at Section 1 due to seismic shear wave propagation.

**Figure 17:**
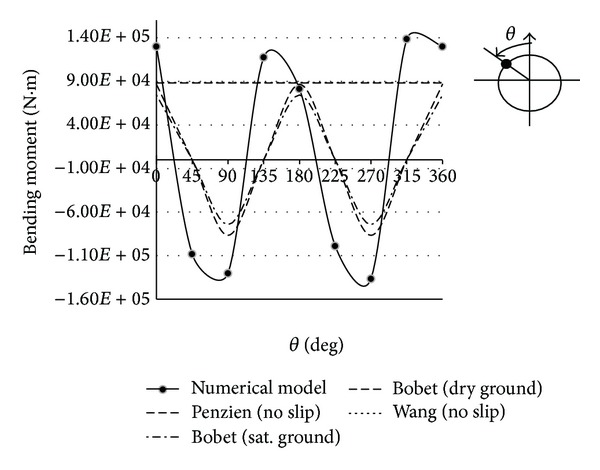
Bending moments at Section 2 due to seismic shear wave propagation.

The bending moment of analytical and numerical methods is compared in Figures 16 and 17. As shown in Figures 16 and 17, one maximum bending moment is obtained by Wang and Penzien methods which is shown by a horizontal line. For seismic ground motions induced by shear waves, maximum and minimum values of bending moment are obtained by Bobet method in dry and saturated conditions, so the results are shown by broken lines.

As shown in Figures 16 and 17 the maximum value of bending moments is obtained by numerical modeling. The results of 4 analytical methods for maximum bending moment are so close and Penzien method has compatible results with other methods. Yet, difference with results of numerical modeling is notable and the values have been shown in Table 6. According to Table 5, the differences of axial forces are less.

## 7. Summary and Conclusion

Underground structures using numerical methods are necessary because of many complicated conditions such as heterogeneous layers, irregular geometry of tunnels, ground water, and soil-structure interactions. The results of numerical methods should be compared with analytical methods. The maximum values of axial forces obtained by numerical analyses are more than the other three analytical methods because of reduction coefficient of shear modulus. The results of analytical methods seemed to be overestimated, since the maximum values of shear modulus have been used.

The result obtained by Bobet method in dry ground is the closest one to the results of numerical analysis in both cross sections. Also the axial forces computed by Bobet method in dry and saturated ground are approximately the same. By assuming saturated condition in Bobet method, the axial forces induced by shear waves are a little less than the axial force obtained in the dry ground. The axial forces computed by Wang method are close to the results of Bobet method. The solutions of Penzien [19] result in values of axial forces are not compatible with the other analytical methods and numerical analysis. On the other hand, the values of axial forces obtained by Penzien method are not rational. This observation was also noted by Hashash et al. [1].

In the case of bending moment, the highest value was obtained by the numerical modeling. The results of four analytical methods are very close to each other, and the results of Penzien method are compatible with the others. However, the differences with the results of numerical modeling are noticeable and Tables 4–6 show these values. The differences of axial forces between analytical methods and numerical modeling are less. So the results show that the axial forces determined using the analytical approaches are in acceptable agreement with numerical analysis results, while the computed bending moments are less comparable and show significant discrepancies.

## Figures and Tables

**Table 1:** Sections specifications used in the analyses.

Section	Tunnel axis level (m)	Groundwater level (m)	Maximum acceleration (g)	Alluvium stratum thickness (m)
1	−22.5	−9	0.36–0.38	39
2	−28.5	−30	0.4–0.42	45

**Table 2:** Geotechnical properties of sections layers.

Section	Layer type	Depth *d* (m)	Density *γ* (kg/m^3^)	Bulk modulus *K* (Pa)	Shear modulus *G* (Pa)	Friction angle *φ* (°)	Cohesion *C* (Pa)
1	Coarse-grained	0–9	1800	18 × 10^6^	18 × 10^6^	35	3 × 10^3^
	Coarse-grained	9–26	2050	18 × 10^6^	18 × 10^6^	35	3 × 10^3^
	Fine-grained	26–39	2050	29 × 10^6^	112 × 10^6^	28	12 × 10^3^

2	Fine-grained	0–7.5	1800	72.5 × 10^6^	24 × 10^6^	27	10 × 10^3^
	Coarse-grained	7.5–15	1850	100 × 10^6^	33 × 10^6^	31	8 × 10^3^
	Fine-grained	15–21	1900	83 × 10^6^	28 × 10^6^	26	14 × 10^3^
	Coarse-grained	21–30	1900	140 × 10^6^	52 × 10^6^	32	8 × 10^3^
	Fine-grained	30–36	2100	140 × 10^6^	52 × 10^6^	30	11 × 10^3^
	Coarse-grained	36–45	2100	140 × 10^6^	52 × 10^6^	32	8 × 10^3^

**Table 3:** Tunnel lining properties.

Cross section *A*_*S*_ (m^2^)	Tunnel radius *r*_0_ (m)	Elastic modulus *E*_*S*_ (GPa)	Poisson's ratio *v*_*S*_	Moment of inertia *I*_*S*_ (m^4^)	Thickness *t* (m)
0.525	4.65	35	0.2	0.005359	0.35

**Table 4:** Maximum values of shear strain and reduced shear modulus.

Section	Shear strain	Shear modulus modification factor
1	0.00058	0.4
2	0.00052	0.36

**Table 5:** Axial forces maximum differences between analytical and numerical methods (%).

Section	Wang and Penzien method	Bobet method (dry medium)	Bobet method (sat. medium)
1	53.1	21.6	32.5
2	54.8	32	54

**Table 6:** Bending moments maximum differences between analytical and numerical methods (%).

Section	Wang method	Penzien method	Bobet method (dry medium)	Bobet method (sat. medium)
1	78	78	60	75
2	53.4	54	58	83
